# Vitamin D and Osteogenesis Imperfecta in Pediatrics

**DOI:** 10.3390/ph16050690

**Published:** 2023-05-03

**Authors:** Francesco Coccia, Angelo Pietrobelli, Thomas Zoller, Alessandra Guzzo, Paolo Cavarzere, Angelo Fassio, Carl-Erik Flodmark, Davide Gatti, Franco Antoniazzi

**Affiliations:** 1 Department of Surgical Sciences, Dentistry, Gynecology and Pediatrics, Pediatric Clinic, School of Medicine, University of Verona, 37129 Verona, Italy; 2 Pediatric Clinic C, Azienda Ospedaliera Universitaria Integrata, 37126 Verona, Italy; 3 Pennington Biomedical Research Center, Baton Rouge, LA 70808, USA; 4 Department of Pathology and Diagnostics, School of Medicine, University of Verona, 37129 Verona, Italy; 5 Department of Medicine, School of Medicine, University of Verona, 37129 Verona, Italy; 6 Department of Clinical Sciences, Faculty of Medicine, University of Lund, 22100 Lund, Sweden; 7 Center for the Diagnosis and Treatment of Rare Skeletal Diseases of the Developmental Age of the Veneto Region, 37126 Verona, Italy

**Keywords:** osteogenesis imperfecta, vitamin D, pediatrics

## Abstract

Osteogenesis Imperfecta (OI) is a heterogeneous group of inherited skeletal dysplasias characterized by bone fragility. The study of bone metabolism, in these disease, is problematic in terms of clinical and genetic variability. The aims of our study were to evaluate the importance of Vitamin D levels in OI bone metabolism, reviewing studies performed on this topic and providing advice reflecting our experience using vitamin D supplementation. A comprehensive review on all English-language articles was conducted in order to analyze the influence of vitamin D in OI bone metabolism in pediatric patients. Reviewing the studies, contradictory data were found on the relationship between 25OH vitamin D levels and bone parameters in OI, and in several studies the baseline levels of 25OH D were below the threshold value of 75 nmol/L. In conclusion, according to the literature and to our experience, we highlight the importance of adequate vitamin D supplementation in children with OI.

## 1. Introduction

Osteogenesis imperfecta (OI), also known as “brittle bone disease”, is a phenotypically and genetically heterogeneous group of inherited skeletal dysplasias characterized by bone fragility, increased risk of fractures and skeletal deformities [1]. In addition to skeletal abnormalities, many other alterations may occur, such as craniofacial and dental abnormalities, blue-grey sclera, joint hypermobility, muscle weakness, cardiovascular and respiratory complications, hearing loss and short stature [2]. 

Osteogenesis imperfecta is a generalized connective tissue disease affecting all tissues expressing type I collagen [3]. Clinical heterogeneity ranges from death in the perinatal period or severely short stature and bone deformities to normal life expectancy with only mild osseous fragility and slightly reduced bone mass [4].

Currently, 22 types of OI have been identified based on clinical and hereditary features and biochemical defects [3]. In most cases, the disease is caused by the pathogenic variants of genes encoding collagen type I, COL1A1 or COL1A2. However, recently discovered forms of OI are not related to molecular defects in the type I collagen genes but are associated with other pathogenic variants in several additional genes that function within the collagen biosynthesis pathway (i.e., impaired collagen synthesis, post-translational modification, secretion and processing) or are involved in osteoblast differentiation or function and bone mineralization [3].

The incidence of OI is approximately 1 in 10–20.000 live births. Although OI is classified as a rare disease, it is one of the most common rare skeletal disorders and causes of genetic bone fragility in children and adolescents [1].

The histomorphometric picture of the bone is usually characterized by the presence of an excessive number of osteoblasts with impaired activity and the consequent deficient deposition of bone matrix [5]. The trabecular skeletal turnover is generally increased and these histomorphometric features have been confirmed through the detection of an increase in bone turnover markers, such as serum osteocalcin, bone alkaline phosphatase, collagen peptide crosslinks (C- or N-telopeptide) and urinary calcium excretion [6,7,8]. 

The increase in bone turnover markers represents the rational for therapy with antiresorptive agents, such as bisphosphonates (BP), which act by inhibiting osteoclast activity and bone resorption and are the mainstay of pharmacological treatment in pediatric patients with OI [9].

The beneficial effect of BP in the treatment of OI has been widely documented. It decreases bone turnover, thus inhibiting osteoclasts, and improves bone mineral density [9]. Dual-energy X-ray absorptiometry (DXA) has been used as a reference method to evaluate therapeutic efficacy over time. There is clear evidence that BPs improve bone microarchitecture, bone mass and long bone deformity and restore vertebral size and shape in OI [10]. However, BP treatment does not improve ligament laxity, and there is limited evidence of a fracture-inhibiting effect for the fractures of long bones [1]. 

Therefore, the effect of BP in OI is less consistent compared to osteoporosis [11], especially in the most severe forms of OI, likely because it does not target the inherent defects in bone quality.

All treatments require adequate vitamin D levels and proper calcium intake, otherwise any treatment is doomed to be ineffective [11].

The most recent meta-analyses of randomized controlled trials suggest a beneficial effect of calcium and vitamin D supplementation on the prevention of fractures [12]. These findings argue for appropriate vitamin D and calcium supplementation in people with high risk of fracture [11].

Vitamin D in humans is produced by skin exposure to sunlight through a conversion of 7-dehydrocholesterol to previtamin D3, which is rapidly converted to vitamin D3 [13]. This vitamin D is metabolized in the liver to 25-hydroxy vitamin D (25OHD), which is used to determine a patient’s vitamin D status [13]. 

Vitamin D is involved in a plethora of biological processes and is an essential component of calcium metabolism that supports bone health and osteogenesis. In particular, it optimizes intestinal calcium and phosphorus absorption for the proper formation of bone mineral matrix and exerts negative feedback on PTH secretion [14].

Currently, there is no clear consensus on the optimal 25OHD levels measured in serum. The threshold for vitamin D deficiency is usually defined as a 25OHD level below 20 ng/mL (50 nmol/L) and that for vitamin D insufficiency as a 25OHD level of 21–29 ng/mL (50–75 nmol/L) [15].

The aim of our study was to evaluate the importance of vitamin D supplementation for bone metabolism in OI and to review the studies conducted on this topic. We also provide some suggestions on the basis of our clinical experience regarding vitamin D supplementation in OI before and during treatment with BP in pediatric patients.

## 2. Results 

Vitamin D deficiency is a worldwide problem in children [16], and in patients with OI, it is also a common issue, with rates comparable to the general pediatric population [17]. Kadhim et al. demonstrated that vitamin D insufficiency and deficiency were found in approximately half of patients with OI. However, the majority of patients with type I OI have adequate vitamin D levels [18]. Many factors contribute to serum vitamin D levels, such as age, weight, height, body mass index (BMI), ethnicity and the consumption of soda, milk and multivitamins [19].

The literature on OI and vitamin D is not extensive from a numerical point of view, and it is diverse in terms of qualitative factors (Table 1). This is due to the fact that it is a rare and very heterogeneous disease in terms of severity and subtypes. Indeed, it is difficult to compare data on bone metabolism between different genotypes and phenotypes of OI. Moreover, the addition of another “confounding factor”, such as vitamin D deficiency, could lead to extremely confounding results [20].

### 2.1. Vitamin D and BMD

Edouard et al. [21] reported a positive correlation between higher vitamin D levels and lumbar spine bone mineral density Z-scores (LS-aBMD) in children and adolescents with OI type I, III and IV. This study found that for every 1 nmol/L increase in serum vitamin D, the LS-BMD Z-score increased by 0.008. 

These conclusions were refuted in another study by the same authors [22], which found no evidence that serum 25OHD levels ranging from 13 to 103 nmol/L were associated with differences in bone mineralization, metabolism or bone mass in children with OI [22].

In another study, Edouard et al. [23] reported insufficient 25OHD serum concentrations (<50 nmol/L) in more than a quarter of 132 patients with OI. More severely affected patients (type III OI) tended to have lower 25OHD levels, higher serum 24,25(OH)2D levels and a higher serum 24,25(OH)2D to 25OHD ratio, suggesting increased 25OHD 24-hydroxylase (CYP24A1) activity [23]. No correlation was found between 24,25(OH)2D and BMD levels, but it can be assumed that these patients with CYP24A1 hyperactivation may require higher doses of vitamin D to achieve sufficient effect on BMD [23].

Wilsford et al. [19] and Zambrano et al. [20] found that a high percentage (greater than 80%) of patients with OI had insufficient or deficient 25OHD levels, confirming the previous findings of Chagas et al. [24]. Bian et al. [25] reported a lower percentage (about 40%) of patients with vitamin D insufficiency in a cohort of 90 OI patients. 

In none of these studies is a clear reference to vitamin D supplementation present, although a significant proportion of the patients studied had reduced levels.

In some studies, with vitamin D supplementation, there is inconsistent information regarding the supplemental vitamin D dose (from 400 IU/day to 2000 IU/day) [25,26,27]. Plante et al. showed that the administration of a higher dose of vitamin D (2000 IU) was not superior to a lower dose (400 IU) for increasing the BMD of the lumbar spine [26].

Patients with OI are often small for their age, weight and height, and this should be considered when adjusting the supplemented vitamin dose to prevent potential adverse effects, such as primary hypercalciuria [2]. Vitamin D intake, leading to overdose or intoxication, and the severity of the corresponding hypercalcemia in patients of pediatric age have not been determined [28].

In order to discriminate the real role of vitamin D on bone metabolism in OI, treatment with BP represents another additional confounding factor. Indeed, it is difficult to understand whether an increase in BMD levels is attributable to Vitamin D supplementation or BP treatment [26]. A study shows how LS-aBMD z-scores increase by about 1.0 in the first year of bisphosphonate treatment [10], but this increase in bone mineral content may not occur if vitamin D levels (and calcium intake) are inadequate [9]. It is therefore possible that a poor response to therapy may be due to a relative insufficiency of vitamin D.

**Table 1:** Studies on Vitamin D in OI: Vit. D levels and Ls-BMD value at baseline in subjects with O.I.

Studies	Type	Patients n; M%; -F%; Age Range (years)	25(OH)D Basal Level nmol/L (±SD)	Bone Mineral Density: LS-aBMD (±z-Score)	Results
Edouard et al. 2011 [21]	Retrospective Cross-sectional Study	n: 315; (M: 51% -F: 49%) 1–18 years	64 (±23)	−3.9 (± 1.6)	Serum 25OH D levels are positively associated with LS-aBMD z-scores
Edouard et al. 2011 [22]	Retrospective study	n: 71 (M: 51%; F 49%) 3–13 years	50 (±18)	−4.6 (±1.3)	No evidence that serum 25(OH)D level was associated with measures of bone mineralization, metabolism or mass in children with OI
Edouard et al. 2012 [23]	Retrospective study	n: 132 (M:52%; F: 48%) 1–18 years	60 (±23)	−4.9 (±1.2)	OI patients with the most severe skeletal phenotype have higher serum 24,25(OH)2D levels and higher serum 24,25(OH)2D to 25OHD ratios, independently of bone mass or bone metabolism
Chagas et al. 2012 [24]	Cross-sectional Study	n: 26 (M: NR%; F: NR%) 13–39 years	65 (±22)	−2.7 (±0.8)	69% and 77% of patients with type I OI and patients with type III OI, presented insufficient vitamin D serum concentrations, whereas 8% of patients with type III OI were considered vitamin D deficient
Wilsford et al. 2013 [19]	Retrospective study	n: 44 (M: 41%; F: 59%) 0–18 years	57 (±27)	Not reported	Almost 80% of children with OI have insufficient or deficient levels of 25OHD
Zambrano et al. 2016 [20]	Cross-sectional study	n: 52 (M: 44%; F: 56%) 0–19 years	54.2 (±11.0)	−1.3 (±0.5)	88.4% of individuals had insufficient or deficient serum 25-OHD levels. Positive association between the BMD z-score of the lumbar spine and serum 25-OHD levels, even after adjusting for sex, age, and OI type
Plante et al. 2016 [26]	double-blind randomized controlled trial	n: 60 (M: 50%; F: 50%) 6–19 years	65.5 (±20.5)	−2 (±1.1)	One-year of RCT children with OI showed that supplementation of either 400 IU or 2000 IU of vitamin D translated into significant increases in serum 25OHD concentrations. However, increases in serum 25OHD concentrations did not have a detectable effect on LS-aBMD z-scores
Bian et al. 2018 [25]	Retrospective	n: 90 (M 50%; F 50%) 4–14 years	82.6 (±29.2)	−0.9 (±1.4)	About 40% of patients with OI have vitamin D insufficiency
Maines et al. 2020 [27]	Prospective	n: 37 (M 54%; F% 46%) 2–20 years	85 (±25)	Not Reported	Low vitamin D levels are to be considered a significant risk factor for post-infusion hypocalcemia after neridronate treatment
Diacinti et al. 2021 [10]	Prospective	n: 60 (M: 58%; F: 42%) 1–16 years	77.3 (±40)	−2.46 (±1.25)	VFA as a safe and alternative methodology in the follow-up of children and adolescents with OI
Mohsenzade et al. 2021 [17]	Case control study	n: 23 (M: 39%; F: 61%) 8 ± 4 years	65 (± 34)	0.47 ± 0.10	Vitamin D deficiency is prevalent amongst OI children in southern Iran

Although there are conflicting data on the relationship between vitamin D levels and bone parameters in OI, it is important to emphasize that in most studies of bone metabolism in OI, baseline vitamin D levels are below the threshold of 75 nmol/L [19,20] (Table 1). The 25OH D levels in OI patients have been inversely associated with PTH concentrations and markers of bone resorption, suggesting a possible deleterious effect of low 25OHD levels on bone [19,20]. This may be a concern because it adds the effects of vitamin D deficiency/insufficiency to the effects of the disease on bone metabolism.

### 2.2. Vitamin D and Calcemia

Maines et al. demonstrated that Vitamin D, in addition to its therapeutic role (i.e., increased calcium absorption, better mineralization), also plays a preventive role in bisphosphonate-induced hypocalcemia [27]. In fact, higher basal vitamin D levels have been shown to reduce the risk of post-infusion hypocalcemia after neridronate treatment in children and adolescents [27] and zoledronic acid in adults [29]. It was found that hypocalcemia was mild and asymptomatic in all cases and calcium levels at baseline and post-infusion (after 24 and 48 h) were related to baseline vitamin D levels, with a positive linear correlation [29].

In this regard, low vitamin D levels should be considered to be a significant risk factor for post-infusion hypocalcemia and should be carefully investigated and treated before neridronate infusion [27]. Vitamin D prophylaxis must be started as soon as possible, given its protective effects, but this recommendation is not always followed [27]. In the study by Maines et al., all patients were advised to take calcium and vitamin D supplementation, although only 38% had insufficient vitamin D values and only 5% had vitamin D deficiency. These data do not differ from those in the literature, which show, on average, the same level of adherence to long-term treatments for chronic diseases in developed countries [27].

## 3. Materials and Methods

The methodology of this systematic review is consistent with the Preferred Reporting Items for Systematic Reviews and Meta-Analyses (PRISMA) guidelines.

A comprehensive search of all English-language articles analyzing the influences of vitamin D in OI bone metabolism in pediatric patients was conducted. 

We searched through PubMed database using the keywords “Osteogenesis Imperfecta”, “Vitamin D”, “children”, to identify relevant studies published between 1 January 1999 and 30 September 2022. The bibliographies of related articles were also examined to identify any additional published references relevant for inclusion in the review.

Search results were exported into the reference manager software “Rayyan QCRI”. Two reviewers (FC and FA), working independently and blindly, considered the potential eligibility of each of the titles and abstracts identified after executing the search strategy. They then evaluated the full-text versions of all potentially eligible studies and extracted data from the references. Disagreements were resolved by a third reviewer (AP). 

Data extracted from each study were: (1) study quality (e.g., study design, outcomes and statistical analyses); (2) study sample characteristics (e.g., age, sex and OI types); (3) study interventions (e.g., vitamin D supplementation); and (4) data on bone mineral density (e.g., lumbar spine bone mineral density). 

Studies were included when they fulfilled the following criteria:

- Study in OI patients in pediatric age,
- Dosing of 25 OH Vitamin D levels,
- Assessment of bone mineral density levels or bone metabolism markers.

**Figure 1:**
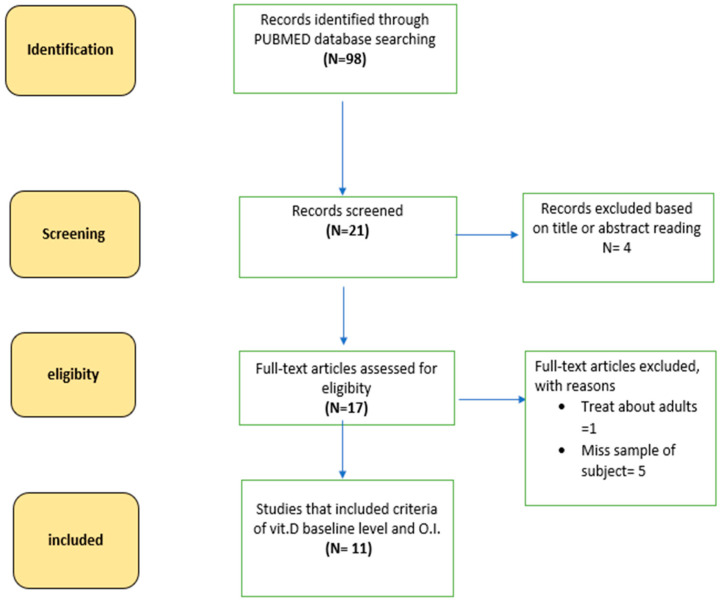
Diagram showing an overview of the study selection process.

The search criteria and the selected articles are shown in Figure 1 and Table 1.

## 4. Discussion and Conclusions

Vitamin D deficiency is a worldwide problem in children and affects one billion people worldwide across all ethnicities and age groups. This deficiency, in absence of supplementation, is common and comparable between OI patients and the general pediatric population, affecting up to 50% of subjects [12,13,14].

The lack of vitamin D should not only be corrected in healthy children but especially in patients with osteogenesis imperfecta.

Vitamin D is a pleiotropic hormone that plays a role in calcium–phosphorus metabolism as well as in the bone health [14,30]. Its proper supplementation in OI helps to prevent the secondary increase in PTH and the following increase in bone turnover [23].

A recent study has also hypothesized a possible positive effect of Vitamin D on preventing scoliosis from worsening in healthy children with idiopathic scoliosis [31]. It would be interesting to evaluate whether patients with OI and scoliosis, a common comorbidity in this population [1,15], may derive a similar benefit from vitamin D supplementation.

In addition, adequate vitamin D levels prevent the occurrence of hypocalcemia, which could be induced by the administration of neridronate [27]. Bisphosphonates play a key role in the treatment of OI. Nevertheless, vitamin D supplementation should be used before starting BP treatment to ensure adequate serum vitamin D levels. It could be suggested that all studies of OI patients should be conducted after achieving sufficient vitamin D serum levels to optimize patient management and to reduce confounding factors.

In our hospital we pay close attention to monitoring vitamin D, calcium and parathormone levels in OI patients. We suggest checking these parameters at least twice a year and performing DXA at least once a year to assess BMD.

To maintain good levels of vitamin D > 75 nmol/L (30 ng/mL), according to the Endocrinology Society’s position, our patients are supplemented with vitamin D, ranging from 1000 to 2000 UI/die based on the severity of the deficiency.

We also recommend a correct oral calcium intake based on patients’ weight, according to Italian Reference Nutrients Values (LARN), to optimize the effect of the vitamin D.

In almost all the reviewed studies [10,20,21,22,26], it was difficult to establish a positive effect of vitamin D on reducing bone turnover markers, such as CTX, ALP and osteocalcin, since most of the patients were treated with BPs.

The correlation between higher levels of vitamin D and BMD seems to not be as linear as suggested by some authors [17,21]. Unfortunately, our review of the literature confirms that vitamin D supplementation is often lacking and not all patients reach desirable levels as is advisable; moreover, we did not find univocal data on the effect of vitamin D on bone turnover markers, such as CTX, ALP and osteocalcin.

Further RCTs studies are needed to establish the real correlation between vitamin D supplementation and BMD or BTM.

## Data Availability

Data sharing is not applicable.

## Abbreviations

OI	Osteogenesis imperfecta
BP	bisphosphonate
ALP	alkaline phosphatase
CTX	terminal c telopeptide
PTH	parathyroid hormone
25OHD	25OH vitamin D
RCT	randomized controlled trials
